# C-reactive protein to albumin ratio as a prognostic tool for predicting intravenous immunoglobulin resistance in children with kawasaki disease: a systematic review of cohort studies

**DOI:** 10.1186/s12969-024-00980-6

**Published:** 2024-04-12

**Authors:** Jue Liu, Xingguang Chen, Minling Yang, Fangfang Shen, Feng Zhu, Jian Jin, Yiqun Teng

**Affiliations:** 1 Department of pediatrics, The Second Affiliated Hospital of Jiaxing University, 314000 Jiaxing, China; 2 Department of orthopedics, The Second Affiliated Hospital of Jiaxing University, 314000 Jiaxing, China

**Keywords:** Kawasaki Disease, C-reactive protein to albumin ratio, Prediction, Intravenous immunoglobulin resistance, meta-analysis

## Abstract

**Background:**

Intravenous immunoglobulin (IVIG) is the primary treatment for Kawasaki disease (KD). However, 10–20% of KD patients show no response to IVIG treatment, making the early prediction of IVIG resistance a key focus of KD research. Our aim is to explore the application of the C-reactive protein to albumin ratio (CAR) for predicting IVIG resistance in children with KD through meta-analysis.

**Methods:**

Cochrane Library, PubMed, MEDLINE, EMbase, CNKI, WanFang, the Chinese Biomedical Database, and CQVIP were searched up to November 2023 for cohort studies on predicting IVIG-resistant KD using the CAR. Articles were selected based on pre-established inclusion and exclusion criteria after extracting literature data and assessing them using the QUADAS-2.0 tool for evaluating the accuracy of diagnostic tests. Stata 15.0 software was used for meta-analysis.

**Results:**

Four Chinese and English literature reports were included in this meta-analysis. The results revealed the presence of a threshold effect and high heterogeneity among the included studies. The combined sensitivity for CAR predicting IVIG-resistant KD was calculated as 0.65 (95% CI 0.58–0.72), specificity as 0.71 (95% CI 0.57–0.81), and the area under the curve (AUC) as 0.70 (95% CI 0.66–0.74) using the random-effects model. The combined positive likelihood ratio was 2.22 (95% CI 1.35–3.65), the combined negative likelihood ratio was 0.49 (95% CI 0.35–0.69), and the diagnostic odds ratio was 5 (95% CI 2–10).

**Conclusion:**

CAR is an auxiliary predictive indicator with moderate diagnostic value that provides guidance in the early treatment of the disease, demonstrating a certain predictive value that warrants further investigation. However, CAR cannot yet be considered as a definitive diagnostic or exclusionary marker for IVIG-resistant KD. Therefore, multi-center, large sample, and high-quality long-term follow-up trials are warranted to confirm the current findings.

## Background

Kawasaki disease (KD) is an acute vasculitis primarily affecting preschool children and predominantly involving small to medium-sized blood vessels. Despite extensive research, the etiology of KD remains unclear [1]. The primary complication associated with KD is the development ofcoronary artery lesions (CALs), with reported occurrence rates ranging from 15 to 25%. In severe cases, CALs may lead to the rupture of coronary artery aneurysms, myocardial infarction, and even sudden death. Remarkably, KD has emerged as the leading cause of acquired heart disease among children [2].

The occurrence rate of CALs can be significantly reduced, from 20 to 25% to approximately 2–4%, by combining high-dose intravenous immunoglobulin (IVIG) therapy with aspirin. This treatment strategy has been currently recognized as the first-line approach [3]. However, a subset of children fails to respond to IVIG, exhibiting persistent fever for more than 36 h after standard treatment or experiencing recurring fever (2–7 days after initial resolution) exceeding 38 °C. This condition, known as IVIG-resistant KD, represents an independent risk factor for CAL development [4]. Administering corticosteroids early on in these patients has been shown to effectively reduce complications and significantly improve prognosis [5, 6]. Therefore, accurate prediction of IVIG-resistant KD at an early stage holds great clinical significance, as it allows for intensified early treatment and improved patient outcomes.

As KD is a vasculitis, blood-based markers can provide some insight into its severity. C-reactive protein (CRP) and albumin (ALB) have been included in various risk scoring systems aimed at predicting IVIG resistance in KD patients due to their presumed association with this phenomenon [7–9]. However, previous studies have demonstrated that using CRP or ALB as single indicators for predicting IVIG resistance is not ideal [10, 11].

Recently, a newly introduced inflammatory parameter called the C-reactive protein-to-albumin ratio (CAR) has shown promise as a more valuable and accurate predictor compared to using CRP or ALB individually. CAR has demonstrated potential in predicting the inflammatory status and prognosis of several clinical diseases [12–14]. Although some studies have investigated the role of CAR in predicting IVIG resistance among KD patients, there is considerable variability in sensitivity (SEN) and specificity (SPE) across these studies, and no systematic review has summarized this evidence to date. Consequently, this study was aimed to collect relevant research results, analyze the data, and conduct a comprehensive meta-analysis to assess the predictive value of CAR in IVIG-resistant KD.

## Materials and methods

### Protocol

The Preferred Reporting Items for Systematic Reviews and Meta-Analyses (PRISMA) Statement was used as a guideline for this study.

### Search strategies

We conducted searches in multiple databases, including the Cochrane Library, PubMed, MEDLINE, EMbase, CNKI, WanFang, the Chinese Biomedical Database, and CQVIP, up until November 2023. The search terms used were combinations of the following keywords: children, pediatric; kawasaki disease; C-reactive protein (CRP); albumin (ALB); and C-reactive protein to albumin ratio (CAR). Both subject and free words were used in the search, and the strategies were finalized after conducting several pre-searches. Additionally, we reviewed the references of the included articles and other relevant studies. There were no language restrictions applied.

### Study selection

According to the Cochrane Collaboration Network’s systematic evaluation manual, the inclusion and exclusion criteria for this meta-analysis were formulated in strict accordance with the participants, interventions, comparators, outcomes, and study design (PICOS) principles.The inclusion criteria were as follows: (1) The research objectives involved conducting a cohort study to evaluate the early diagnostic value of CAR in IVIG-resistant KD; (2) The study subjects were KD patients who met the relevant diagnostic criteria; (3) Four-fold table data could be directly obtained from the literature or calculated; (4) The sources of reagents and detection methods were clearly defined, and the detected specimens were serum.

The exclusion criteria were as follows: (1) Descriptive studies and case-control studies; (2) Data that was not completely provided or was difficult to extract; (3) Repeated reports (inclusion or exclusion depending on the quality of the literature), reviews, conference papers, or only abstracts where the full text was not available.

### Data extraction and quality assessment

For each study, two authors (Jue Liu & Xingguang Chen) independently extracted data and assessed quality. Any disagreements were resolved by the corresponding author (Yiqun Teng & Jian Jin). A standardized data extraction form was used, including:1) Baseline characteristics of the literature: publication date, country or region, number of cases, age, study type, time span, diagnostic criteria, the cut-off value of CAR; (2) Information on diagnostic test parameters: True positive (TP), false positive (FP), false negative (FN), and true negative (TN).

The included studies underwent quality assessment using the Quality Assessment of Diagnostic Accuracy Studies 2.0 (QUADAS-2.0) [15]. Each item in the included studies was evaluated as “yes”, “no”, or “unclear”. A score of 1 was assigned for “yes,” and 0 for “no” or “unclear.” The literature was considered of higher quality if its score exceeded 7.

### Statistical analysis

Stata 15.0 software (StataCorp LLC, North Carolina, USA) was used for statistical analysis. The heterogeneity of the included studies was assessed using the Q test and *I*^*2*^ index. If no heterogeneity (*p* > 0.1 or *I*^*2*^ *<* 50% for Q test) was detected for SEN, SPE, positive likelihood ratio (PLR), negative likelihood ratio (NLR), diagnostic odds ratio (DOR), a fixed-effects model based on the Mantel-Haenszel method was used for pooling. Additionally, the summary receiver operator characteristic (SROC) curve was plotted, and the area under the curve (AUC) was calculated. Otherwise, if there was heterogeneity, the random-effects model using the DerSimonian-Laird method was applied. Clinical utility assessment was conducted using Fagan’s nomogram. Publication bias was assessed using Deek’s funnel plot. Bivariate boxplots were employed to identify literature with high heterogeneity. Cohen’s Kappa analysis was used for the diagnostic consistency analysis.

## Results

### Description of studies

**Fig. 1:**
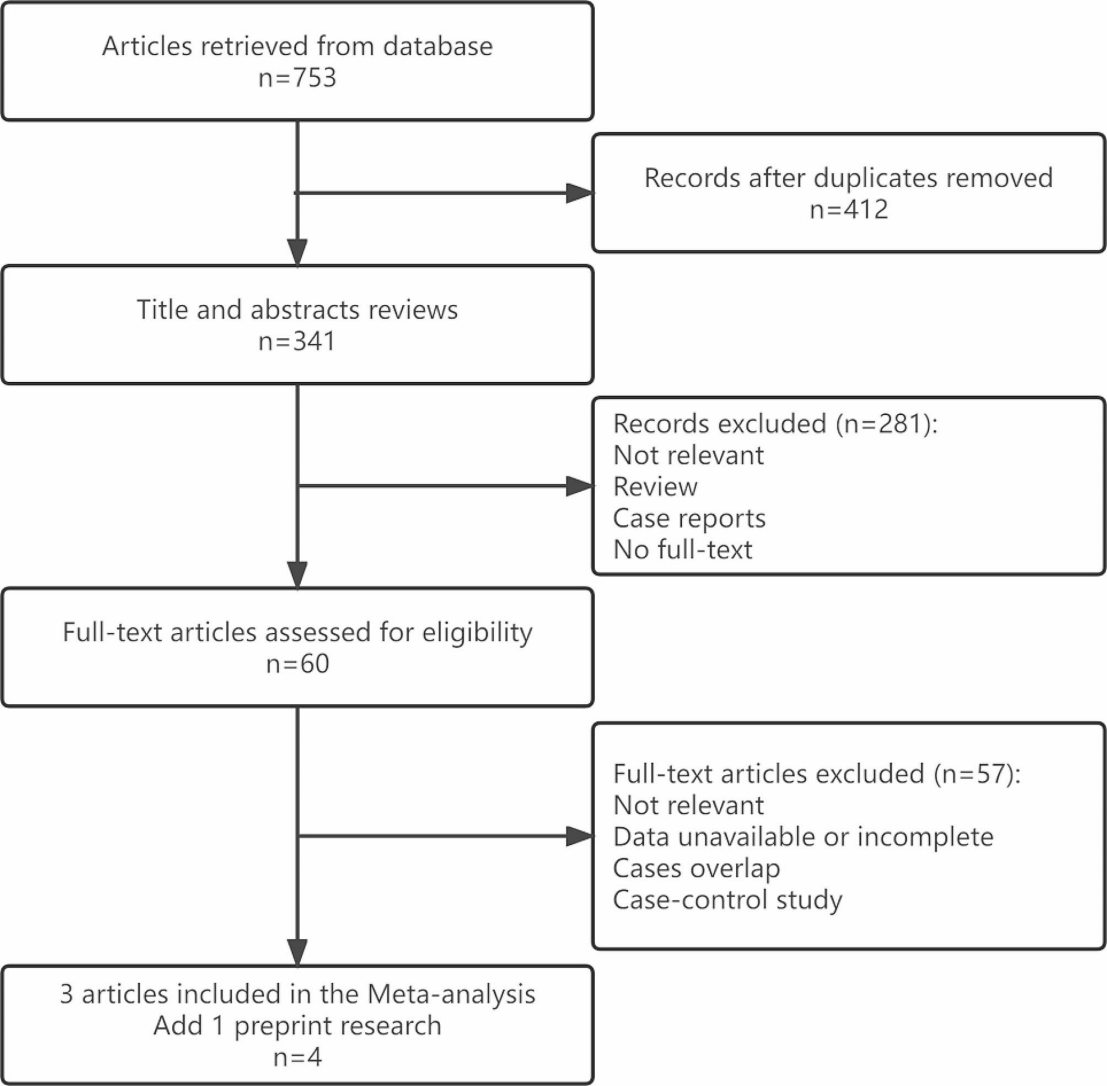
Flowchart of the paper screening process

A total of 753 articles were retrieved from all databases. After carefully reviewing the title and abstract, 693 articles were excluded. The full text of the remaining 60 abstracts was thoroughly analyzed. Among them, 57 papers were found to be incomplete, lacking available outcome data or containing population repetition. Ultimately, four studies [16–19] were included for the final review, with one from the preprint database (Fig. 1).

**Fig. 2:**
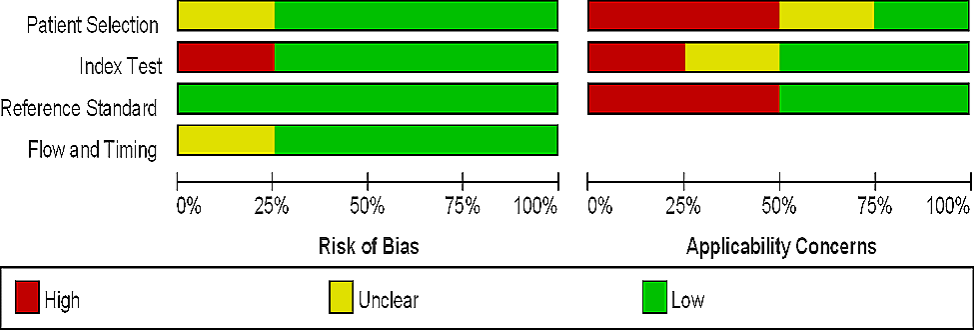
Quality assessment of the included studies

Two prospective cohort studies were included in this meta-analysis. All the research conducted on KD patients was carried out in China, and the diagnostic criteria followed the guidelines set by the American Heart Association (AHA). A total of 2326 cases of KD patients were involved in this meta-analysis (Table 1). The quality of the included articles was assessed using QUADAS-2.0, indicating a medium to high level of quality as illustrated in Fig. 2.

**Table 1:** Baseline analysis of the 4 included papers

Study	Year	Region	Study design	Study period	Diagnostic standard	Study Group	Gender (n)		Age (mean [SD], month)	Cut-off value	TP (n)	FP (n)	FN (n)	TN (n)
							Male	Female						
Li Gang [16]	2020	Sichuan, China	RCS	2013.06-2019.08	AHA	IVIG response	486	312	23.0 (12.0–38.0)	3.14	111	198	48	600
						IVIG-resistance	84	75	26.0 (13.0–40.0)					
Liu Xiaoliang [17]	2021	Sichuan, China	PCS	2015.03-2019.06	AHA	IVIG response	271	200	24.0 (13.0–42.0)	2.07	48	211	31	260
						IVIG-resistance	43	36	28.0 (14.0–54.0)					
Wang Zhenquan [18]	2021	Zhejiang, China	RCS	2021.01-2022.12	AHA	IVIG response	653		17.4	2.20	36	254	25	399
						IVIG-resistance	61		24.4					
Wu Hang [19]	2023	Zhejiang, China	PCS	2018.01-2019.12	AHA	IVIG response	47	48	28.0(14.0–51.0)	2.89	5	12	5	83
						IVIG-resistance	5	5	31.0 (14.0–71.0)					

RCS: Retrospective cohort study; PCS: Prospective cohort study; AHA: American heart association; TP: True positive; FP: false positive; FN: false negative; TN: true negative; IVIG: Intravenous immunoglobulin; SD: Standard deviation.

### Publication bias and SEN analysis

Publication bias was assessed through Deeks’ funnel plot. The plot showed that the four studies included in the analysis were evenly distributed on both sides of the regression line. The Bias *t*-test (*t* = -0.38, *p* = 0.740 > 0.05) indicated that there was no significant publication bias among the included literature. SEN analysis was performed by sequentially excluding each study that was included, and no significant changes were observed. Therefore, the combined results were robust, and the impact of publication bias was minimal.

### Results of meta-analysis

#### Summary analysis

**Fig. 3:**
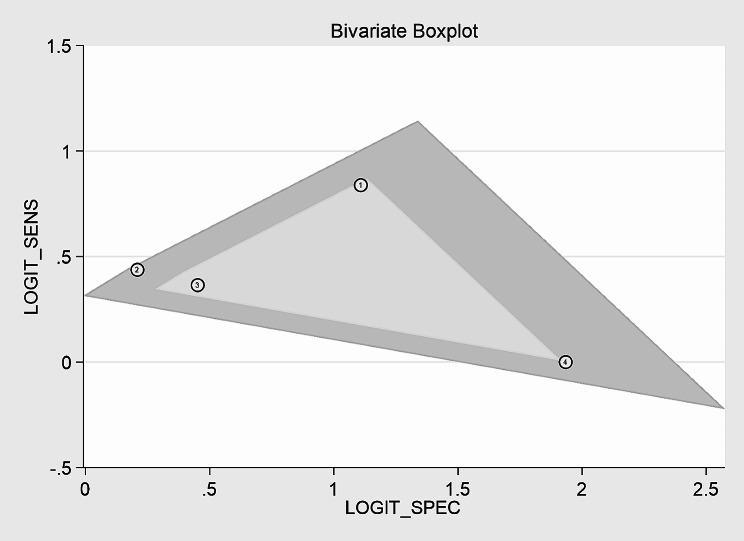
The result of heterogeneity analysis by bivariate boxplot

**Fig. 4:**
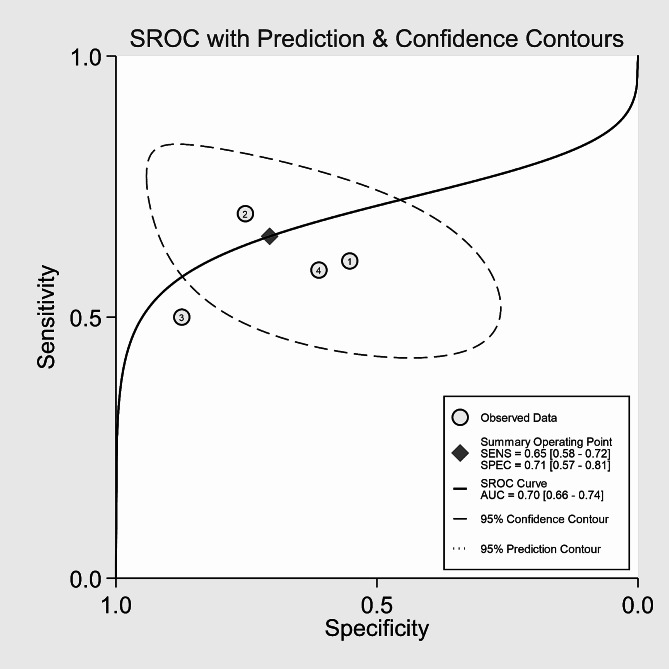
SROC curve of CAR for predicting IVIG-resistant children with KD

The SEN of the CAR for predicting IVIG-resistant KD ranged from 50.0 to 70.0%, while the SPE ranged from 54.8 to 87.0% in the four included studies. The analysis of heterogeneity results (*I*^*2*^ = 61%, *p* = 0.038 < 0.05) indicated a certain degree of heterogeneity. Additionally, the results of the bivariate boxplot also suggested the presence of heterogeneity (Fig. 3). These findings indicate a significant threshold effect in the included literature as evidenced by the “arm-shoulder” distribution observed in the SROC curve (Fig. 4), consistent with the results of Spearman correlation analysis (*ρ* = 0.400, *P* = 0.600 > 0.05). The results of Cohen’s Kappa analysis (k = 0.176) indicated low diagnostic consistency and poor agreement.

### Predictive accuracy of CAR

**Fig. 5:**
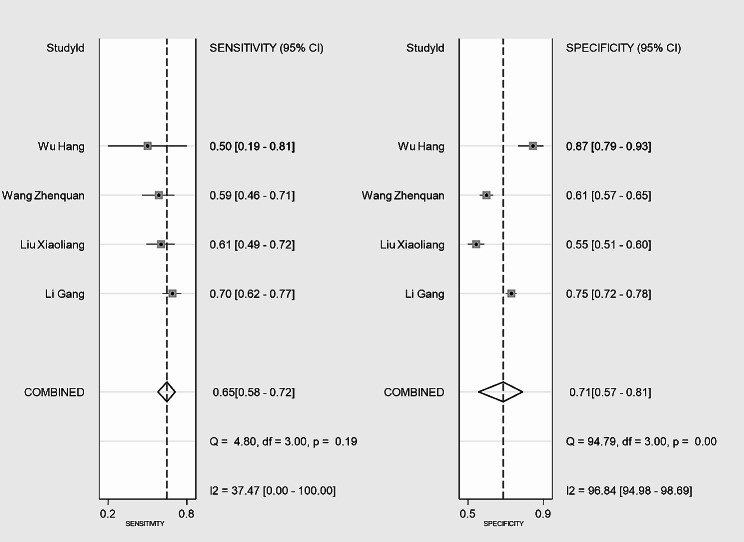
The SEN and SPE of CAR for predicting IVIG-resistant children with KD

**Fig. 6:**
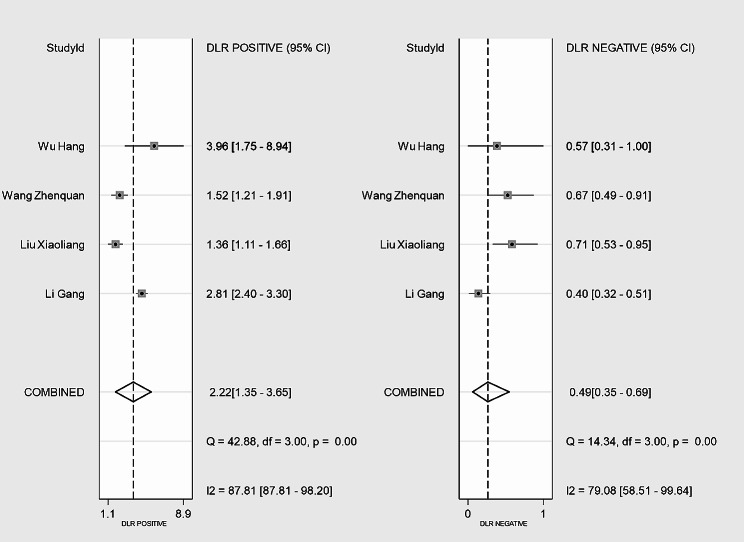
The PLR and NLR of CAR for predicting IVIG-resistant children with KD

**Fig. 7:**
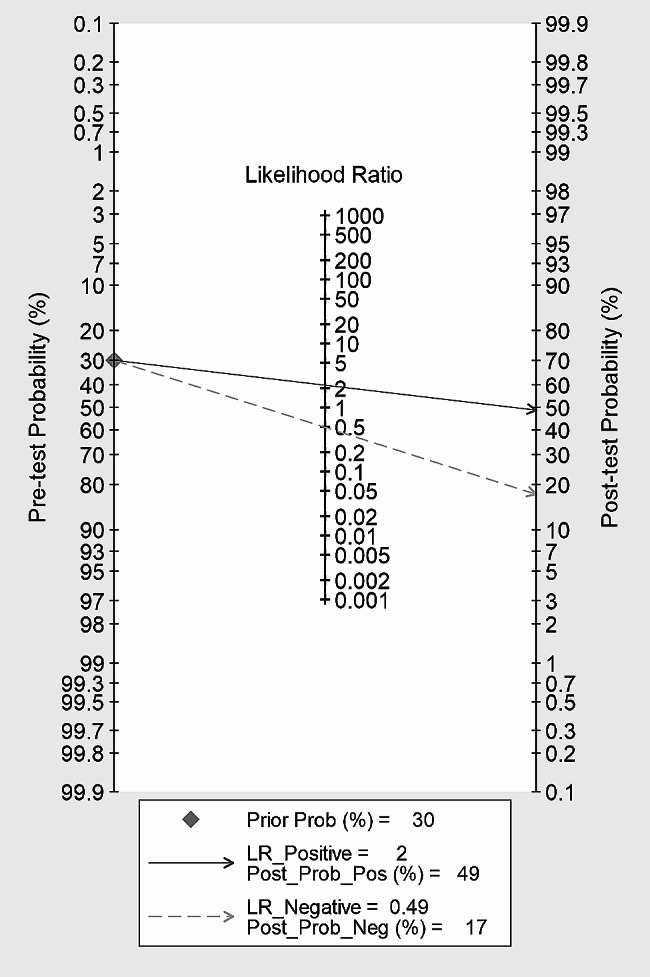
Fagon’s plot of CAR for predicting IVIG-resistant children with KD

Pooled SEN and SPE of CAR for predicting IVIG-resistant KD were 0.65 (95% *CI*: 0.58–0.72) and 0.71 (95% *CI*: 0.57–0.81), respectively (Fig. 5). These results showed that CAR had a certain ability to discriminate children with IVIG-resistant KD. The pooled PLR was 2.22 (95% *CI*: 1.35–3.65), and the pooled NLR was 0.49 (95% *CI*: 0.35–0.69) (Fig. 6). These findings indicated that CAR could be used as an additional indicator for diagnosis, but not as a definitive diagnostic tool or exclusion criterion in IVIG-resistant KD. The AUC was 0.70 (95% *CI*: 0.66–0.74) (Fig. 4), and the DOR was 5 (95% *CI*: 2–10). These results suggested that CAR had a moderate predictive value for IVIG-resistant KD. CAR might hold clinical value in predicting IVIG-resistant KD. As shown in Fig. 7, when CAR is above the cut-off value, the likelihood of IVIG resistance in KD patients increases from 20 to 36%; conversely, it decreases to 11%.

## Discussion

Recently, several studies have investigated the predictive ability of serum inflammatory markers, such as the platelet-to-lymphocyte ratio, neutrophil-to-lymphocyte ratio, CRP, ALB, and CAR, in determining the resistance to IVIG treatment in patients with KD [20–22]. Chaudhary H et al. [23] and Kaya Akca U et al. [24] conducted comprehensive reviews on potential biomarkers for KD, comparing ten different systems for predicting IVIG resistance. The results of their studies indicated that the predictive ability of these indicators in determining whether KD patients would develop IVIG resistance is unsatisfactory. Although Liu C et al. [25] performed a meta-analysis on the literature regarding the indicators of PLR, NLR, and their combination for IVIG-resistant KD, they did not analyze the role of CAR.

Our recent studies [19, 26] have found that CAR demonstrates good predictive capabilities for IVIG resistance and CAL. However, it lacks robust evidence from evidence-based medicine. Therefore, we conducted this meta-analysis with the aim of exploring the ability of CAR to predict IVIG resistance in patients with KD and providing evidence for CAR as an independent predictor or its inclusion in new predictive scoring models, so as to enhance early therapeutic efficacy for KD patients.

This meta-analysis included four studies on CAR predicting IVIG resistance in patients with KD. The pooled results indicated that CAR demonstrated a certain ability to predict IVIG resistance in KD patients (SEN: 0.62, SPE: 0.71). However, at present, CAR can only serve as an auxiliary predictive indicator for the occurrence of IVIG resistance and cannot be used as a definitive diagnostic or exclusionary marker in KD (PLR: 2.22, NLR: 0.49). Overall, CAR has a moderate predictive value for the occurrence of IVIG resistance in KD patients. It was further confirmed by the results of Fagan’s plot.

Therefore, currently, we cannot determine whether CAR can serve as the preferred independent predictive biomarker for predicting IVIG resistance in patients with KD. Establishing a new clinical prediction model that integrates CAR, NLR, and PLR may be an important approach for early prediction of IVIG resistance in KD patients [27]. This is also the current research direction of our research team.

Combining the results of the QUADAS-2.0 quality assessment, we analyzd possible sources of heterogeneity: (1) Though our study used a random-effects model to minimize the impact of threshold effects, different cut-off values will directly influence the research outcomes; (2) CRP and ALB showed differences in various aspects, such as age, gender, and disease duration [28]. The ratio between them might amplify or diminish these differences. Expanding the sample size and determining cut-off values based on age or gender are also directions for future CAR research; (3) Search strategies, publication bias, and gray literature might be significant sources of heterogeneity. We have made efforts to obtain gray literature through various means, such as Wang et al. [18], to minimize heterogeneity. Reducing heterogeneity might have a potential in enhancing the credibility and accuracy of new meta-analyses.

In addition to the high heterogeneity, there were other limitations in this review: (1) All included studies were conducted on Chinese individuals, which might affect the generalizability of the conclusions; (2) There has been a lack of original literature on CAR, requiring further research to draw more definitive conclusions. However, despite these limitations, this meta-analysis alsohad certain strengths. It was the first study to explore the predictive efficiency of CAR in KD patients with IVIG resistance through meta-analysis. The results of our meta-analysis supported CAR as a significant indicator for predicting IVIG resistance in KD patients, thus warranting further in-depth investigation.

## Conclusion

In conclusion, our study may contribute to further improving the clinical management of IVIG-resistant KD. The research findings indicated that CAR might bean additional predictive indicator with moderate diagnostic valueto guide early treatment of the disease, demonstrating a certain predictive value for further investigation. However, CAR cannot be considered a definitive diagnostic or exclusionary marker for IVIG-resistant KD at this time. Therefore, multi-center, large sample, and high-quality long-term follow-up trials are warranted to confirm the current findings.

## Data Availability

The raw data supporting the conclusions of this article will be made available by the authors, without undue reservation.

## Data availability

All data are included in this published article.

## Abbreviations

IVIG: Intravenous immunoglobulin
KD: Kawasaki disease
CAR: C-reactive protein to albumin ratio
CALs: Coronary Artery Lesions
CRP: C-reactive protein
ALB: Albumin
SEN: Sensitivity
SPE: Specificity
PRISMA: Preferred Reporting Items for Systematic Reviews and Meta-Analyses
TP: True positive
FP: False positive
FN: False negative
TN: True negative
PLR: positive likelihood ratio
NLR: negative likelihood ratio
DOR: diagnostic odds ratio
SROC: Summary receiver operator characteristic
RCS: Retrospective cohort study
PCS: Prospective cohort study
AHA: American heart association
PLR: platelet-to-lymphocyte ratio
NLR: neutrophil-to-lymphocyte ratio
